# Study of Thermo-Sensitive *In-Situ* Gels for Ocular Delivery

**DOI:** 10.3797/scipharm.1010-04

**Published:** 2011-03-05

**Authors:** Manas Bhowmik, Sanchita Das, Dipankar Chattopadhyay, Lakshmi K. Ghosh

**Affiliations:** 1 Pharmaceutics Research Lab II, Department of Pharmaceutical Technology, Jadavpur University, Kolkata-700 032, India; 2 Department of Polymer Science & Technology, University College of Science & Technology, 92, A.P.C. Road, University of Calcutta, Kolkata-700 009, India

**Keywords:** Methylcellulose, Hydroxypropylmethylcellulose, *In-situ* gel, ORS

## Abstract

The aim of the present study was the development of thermo-sensitive *in-situ* gels for *in-vitro* evaluation of ophthalmic delivery systems of ketorolac tromethamine (KT), based on methylcellulose (MC) in combination with hydroxypropylmethyl cellulose (HPMC). The gel temperature of 1% MC solution was observed at 60°C. It was found that 6% oral rehydration salt without dextrose (ORS) was capable to reduce the gel temperature below physiological temperature. HPMC was added to increase viscosity and drug release time. The results indicated a large increase in viscosity at 37°C with addition of HPMC whch provided sustained release of the drug over a 4h period. From *in-vitro* release studies, it could be concluded that the developed systems were thus a better alternative to conventional eye drops.

## Introduction

The conventional liquid ophthalmic formulations are washed out from the precorneal area immediately upon instillation because of constant lacrimal secretion, nasolacrimal drainage and short precorneal residence time of the solution [1]. As a result, frequent instillation of solution or higher drug concentration is needed to achieve the desired therapeutic response [2, 3]. But this attempt is potentially dangerous if drug solution drained from the eye is systemically absorbed from the nasolacrimal duct [4]. To increase precorneal residence time and ocular bioavailability, different ophthalmic delivery systems such as viscous solutions, ointments, gels, suspensions or polymeric inserts are used [5]. But because of blurred vision (e.g. ointments) or lack of patient compliance (e.g. inserts), these formulations have not been widely accepted. This problem can be overcome by using *in-situ* gel forming ocular drug delivery system, prepared from polymer, exhibit sol-to-gel phase transition due to a change in a specific physico-chemical parameter (pH, temperature, etc.) in their environment [6] and as a result sustained drug release to the eye occurs [7]. Such formulations where used to delivered bioactive agents by instillation into the eye, which upon exposure to the eye temperature changes to the gel phase [8]. Thus precorneal residence time of the delivery system is increased and ocular bioavailability is also enhanced. MC solution is known to undergo thermoreversible sol-to-gel transition. Gel temperature of MC can be reduced by adding salts and other additives [9].

The objective of the research work was to formulate thermo-sensitive *in-situ* gel ocular delivery systems. The model drug used was ketorolac tromethamine, to treat seasonal allergies such as itching, swelling and inflammation of the eyes.

## Results and Discussion

### In-vitro gelation studies

**Fig. 1.**
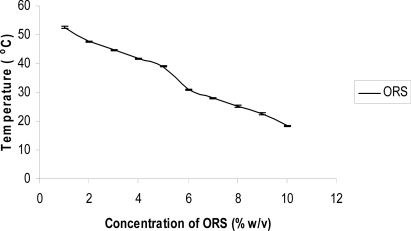
Variation in gelation temperature of 1% MC solution with increasing concentration of ORS (mean ± S.D., n=3).

The gel temperature of 1% MC solution is 60°C. The effect of ORS on gel temperature of MC solution has been shown in figure 1. The gel temperature is reduced below body temperature by addition of ORS. The addition of salt will affect the structure of water, which is mainly due to the interactions between ions and water molecules. Salting out salts of ORS stronger interact with water than hydrogen bonds between water molecules. As a result the hydrogen bonds between water molecules are destroyed by the salt [10]. Salt ions also attract more water molecules due to their stronger hydration abilities than MC chains. This effect decreases water-solubility of MC. In this situation formation of hydrophobic aggregates are more pronounced in a salt containing MC solution. The increased salt content results in fewer free water molecules around MC chains and a stronger hydrophobic environment for MC. So the sol-gel transition occurs at a lower temperature. It has been observed that 6% ORS is capable to ensure a gelation temperature below body temperature and the solutions are free flowing liquid to allow reproducible instillation into the eye as drops at room temperature. HPMC does not significantly alter the gel temperature. All the developed formulations were evaluated for clarity by visual inspection and clarity was sufficient.

### Viscosity study

The viscosity of the formulations is investigated as a function of temperature. The formulations behave as liquids and exhibit low viscosity at 25°C. At 37°C, the solutions are converted into gels with high viscosity. The reasons for this increase in viscosity are that the polymers are fully hydrated and simple entanglement exists between polymer chains at low temperature. The hydration of polymer by water is gradually weakened when the temperature increases, the hydrophobic association of polymer becomes more pronounced and a gel structure is formed. It has been observed that the viscosity enhancing capability of HPMC is much higher than MC alone as shown in table 1.

### Swelling study

The *in-situ* gels were observed to be stable throughout the period of swelling (7 hr.). The rate of swelling of F2 was slower than that of F1 (Table 1). This is due to the higher viscosity of F2 at 37°C which decreases water penetration. The results show that HPMC containing *in-situ* gels are less swollen than MC gel because of formation of highly stiff gels of HPMC in combination with MC.

### In-vitro drug release studies and release kinetics

**Fig. 2.**
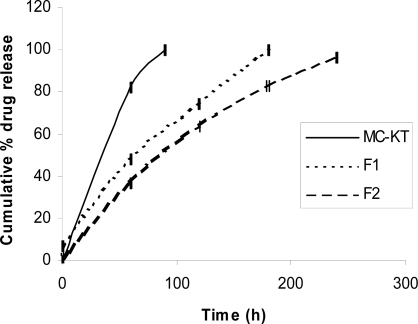
Effects of polymers and ORS on *in-vitro* drug release from MC-Ketorolac solution and *in-situ* gel (mean ± S.D., n=3).

According to the *in-vitro* drug release profiles of *in-situ* gels (figure 2), the drug loading is nearly fully released within 1.5 hrs from MC solution. At 37°C, the *in-situ* gel matrix is formed in which water penetration and hydration is the rate limiting step of drug release. It has been observed that addition of ORS (mixture of 3.5g NaCl, 1.5g KCl and 2.5g NaHCO_3_) will increase the drug release time up to 3hrs. This is due to an increase in viscosity of *in-situ* MC gel solution in presence of ORS at 37°C. This leads to slower solvent penetration and consequently the drug release time will be increased. It is also observed that the drug release time increases up to 4 hours with the addition of HPMC in MC solution at MC/HPMC ratio 2:1, and this increase in drug delivery time is due to increase in viscosity of MC/HPMC combination.

Zero-order, First-order and Higuchi’s equation were employed to interpret the drug release patterns of *in-situ*-gels. It was found that the *in-vitro* drug release pattern is best explained by Higuchi’s equation, as the plots show the highest linearity (*r^2^* *_>_* 0.9884±0.0011) followed by first order and zero order equation (Table 1). The results show that viscosity of F2 is higher than F1 and increased viscosity causes slower drug release. It has been observed that the drug release rate is decreased by addition of HPMC. All the kinetic data were analyzed by the Korsmeyer-Peppas Equation (M_t_/M_¥_=kt^n^). An acceptable linearity (*r^2^* > 0.9963±0.0039) was observed and the release exponent ‘n’ varied from 0.51±0.01 to 0.78±0.06, which indicated a non-Fickian drug diffusion i. e., coupled diffusion and erosion mechanisms.

### In-vitro compatibility studies

In human the average tear volume is 6.5–10.7μl/minute and the tear flow is 0.5–2.2 μl/minute [11]. The hydration rate of the *in-situ* gels as depicted in table 1 is lower than the normal tear flow rate. NaCl, KCl and NaHCO_3_ show iso-osmolarity to the body fluid at 154 mmol, 160 mmol and 175 mmol respectively [9]. According to the results in table 1 the amounts of leaked salts are negligible. In addition, 0.5–2% (85.5–342 mmol) NaCl is tolerable to normal eye [12]. The results show that the developed *in-situ* gels are compatible to the eye and there are no problems to expect due to irritation of eye caused by excessive dehydration of aqueous ocular cavity.

### Stability study

The stability study was carried out on the formulation at 30 ± 2°C and 60 ± 5% RH over the period of 90 days. The formulations retained clarity and no remarkable changes were observed in gelation temperature, viscosity, *in-vitro* drug release profile and salt leaching properties as shown in table 2.

## Experimental

### Materials

Metolose SM 4000 (Methylcellulose, 29.6% methoxyl content) was obtained from Shinetsu Chemical Co. Ltd., Japan. Hydroxypropylmethylcellulose (HPMC K15 M) from Colorcon Asia Pvt. Ltd., Verna, Goa, India and Ketorolac tromethamine from Sun Pharma, Baroda, Gujrat, India were gift samples. The dialysis membrane (LA390, average flat width-25.27 mm, average diameter-15.9 mm and capacity approx-1.99 ml/cm) was purchased from HiMedia Laboratories Pvt. Ltd., Mumbai, India. Sodium chloride (NaCl), Potassium chloride (KCl), Calcium chloride (CaCl_2_) and Sodium bicarbonate (NaHCO_3_) were purchased from E. Merck India Pvt. Ltd., Mumbai, India. All other chemicals were analytical grade.

### Preparation of sample solutions

The methylcellulose (MC, 1%) was dispersed in hot deionised water of 70°C and then cooled down below 20°C under continuous stirring until a homogenous dispersion was obtained [13]. The dispersion was kept in a refrigerator for 48 hours to get clear solution. Oral rehydration salt without dextrose (ORS: mixture of 3.5g NaCl, 1.5g KCl and 2.5g NaHCO_3_) was dissolved in the MC solution and evaluated for gel temperature in order to identify the compositions suitable for *in-situ* gel systems. Ketorolac was then added to the *in-situ* gel systems and the effect of KT on gel temperature was evaluated. The HPMC solution (1%) was prepared by same procedure like the MC solution. The required amount of MC and HPMC solutions were mixed homogeneously to prepare MC-HPMC solutions at a ratio of 2:1 and its *in-situ* gel solution was also prepared by adding ORS and KT.

### In-vitro gelation studies

The gelation studies were carried out with a cell, equipped with a thermo jacket for maintaining constant temperature. The cell was a cylindrical reservoir capable of holding 3 ml of gelation solution. This gelation solution was artificial tear fluid (composition: 0.67g NaCl, 0.20g NaHCO_3_, 0.008g CaCl_2_.2H_2_O and distilled water qs to 100g) [14]. The reservoir was filled with 2 ml of the gelation solution. A 250μl transparent plastic cup present in the bottom of the cell to hold the gel sample in place, after its formation. 100 μl of the formulated solution was injected into the cavity of the cup, with a thermal cycle of 20°C to 70°C. The gel temperature was recorded by visual inspection repeatedly [15]. The temperature was verified with test tube tilting method by observing the non flowing state of the solution [6].

### Rheological studies

The rheological studies were conducted in a viscometer (TV-10 Viscometer, Toki Sangyo Co. Ltd., Japan). The viscosity of the formulated solutions was measured at a shear rate of 20 rpm at 37°C. The temperature was maintained by a thermally controlled water bath. The samples were equilibrated for 10 minutes to reach the running temperature prior to each measurement.

### Swelling study

The studies were conducted with a cell, equipped with thermo jacket to maintain constant temperature. The cell containing artificial tear fluid was used as swelling medium equilibrated at 37°C. The 1 ml of formulated solution was packed in a dialysis bag and put into the swelling medium. At specific time intervals the bag was removed from the medium and weight was recorded. The swelling of the polymer gel as a function of time was determined by using the following relationship [16]: % S_t_ = (W_t_–W_0_)×100/W_0,_ where S_t_ is swelling at time‘t’, W_0_ is the initial weight of the gelling solution and W_t_ is the final weight of the gel.

### In-vitro drug release studies and release kinetics

The release of ketorolac tromethamine from *in-situ* gels was determined by using a Franz diffusion cell. The dissolution medium was artificial tear fluid (ATF). The dialysis membrane, previously soaked overnight in the dissolution medium, was mounted on one end of the donor cylinder of the Franz diffusion cell. The acceptor compartment cylinder was filled with 40 ml of dissolution medium of 37°C so that the membrane just touched the medium surface and the stirring rate was maintained at 50 rpm. One milliliter of the formulation was placed over the dialysis membrane. Aliquots, of 1 ml each were withdrawn at hourly intervals and replaced by an equal volume of fresh medium. The ketorolac tromethamine content of the aliquots was determined by UV spectrophotometry at 323nm.

### In-vitro compatibility studies

The studies were comprised assessment of gel hydration and salt leaching properties of *in-situ* gel. As generally one or two drops of solution are instilled into the eye, the hydration rate of formulations was calculated according to the following equation: Hydration rate [μl/minute] = (9.9122 × W_gs_ × S_t_ × 0.05)/t_m_. Where W_gs_ is the weight of 1 ml of gel solution [gm], S_t_ is the swelling percentage at time t, t_m_ is the swelling time [min] and 100 gm ATF was approximately 99.122 ml in volume. The hydration rate of the formulations derives from the above equation was compared with the average tear flow rate of humans. The salt leaching properties of the formulation were performed similar to the methods for *in-vitro* drug release studies. The aliquots were subjected to examine by Easy Lyte Na/K Analyzer (Medica, USA).

### Stability study

The formulation F2 showing optimum gelation, viscosity and drug release was selected for the stability study which was conducted according to the International Conference on Harmonization guidelines, 2003. A sufficient quantity of gel solution in glass vials was stored in desiccator’s containing saturated solution of sodium chloride to maintain an approximate relative humidity of 60 ± 5%. The desiccator was kept at room temperature (30 ± 2°C) and samples were withdrawn at 0, 30, 60, 90 days. The physical stability of the gel was inspected periodically by checking clarity, gel temperature, viscosity, *in-vitro* drug release profile and salt leaching properties.

## Figures and Tables

**Tab. 1.** Characteristics and *in-vitro* drug release kinetics of *in-situ* gel systems (n=3).

**Formulation**	**Composition (1% Polymer solution + 0.5% KT + 6% ORS)**	**Viscosity at 20 rpm and 37°C (cP± S.D.)**	**Swelling at 3 hrs. (K± S.D.)**	**Hydration Rate of Each Drop (0.05 ml) of** ***in-situ*** **Gel (μl/min)**	**Leached ion Concentration (mmol min^−1^** **drop^−1^** **× 10^−4^)**	

	**MC:HPMC**				**Na^+^**	**K^+^**
F1	1:0	15100.00 ± 6.24	0.04 ± 0.00	0.0097 ± 0.0008	2.17 ± 0.06	0.30 ± 0.02
F2	2:1	17200.00 ± 4.73	0.03 ± 0.00	0.0119 ± 0.0001	1.58 ± 0.08	0.17 ± 0.01

	**Zero-order**	**First-order**	**Higuchi**	**Korsmeyer-Peppas**		

	**r^2^** **± S. D.**	**r^2^** **± S. D.**	**r^2^** **± S. D.**	**r^2^** **± S. D.**	**n**	

F1	0.9791 ± 0.0012	0.9877 ± 0.0009	0.9903 ± 0.0012	0.9965 ± 0.0009	0.51 ± 0.01	
F2	0.9708 ± 0.0004	0.9818 ± 0.0073	0.9884 ± 0.0011	0.9963 ± 0.0039	0.78 ± 0.06	

**Tab. 2.** Stability profiles of F2 *in-situ* gel (n=3)

**Time interval (Days)**	**Gel temperature (°C) (mean ± S. D.)**	**Viscosity (cP) at 20 rpm and 37°C (mean ± S. D.)**	**Cumulative % drug release at 4h (mean ± S. D.)**	**Leached ion Concentration (mmol min^−1^drop^−1^×10^−4^)**	
				**Na^+^**	**K^+^**
0	31.03±0.25	17200±4.75	96.76±0.37	1.58±0.08	0.17±0.00
30	31.10±0.20	17205±6.51	97.75±0.73	1.54±0.06	0.18±0.01
60	31.17±0.32	17202±6.81	96.20±0.32	1.53±0.04	0.16±0.01
90	31.13±0.45	17207±9.61	97.23±0.91	1.55±0.05	0.18±0.00
